# Astaxanthin improves assisted reproductive technology outcomes in poor ovarian responders through alleviating oxidative stress, inflammation, and apoptosis: a randomized clinical trial

**DOI:** 10.1186/s13048-024-01537-7

**Published:** 2024-10-31

**Authors:** Anahid Shafie, Ashraf Aleyasin, Mojtaba Saffari, Mojtaba Saedi, Sahar Rostami, Saeede Rezayi, Seyed Danial Mohammadi, Fardin Amidi

**Affiliations:** 1 Department of Anatomy, School of Medicine, Tehran University of Medical Sciences, Poorsina Ave, P.O. Box: 1461884513, Tehran, Iran; 2 Department of Infertility, Shariati Hospital, Tehran University of Medical Sciences, Tehran, Iran; 3 Department of Obstetrics and Gynecology, Shariati Hospital, Tehran University of Medical Sciences, Tehran, Iran; 4 Department of Medical Genetics, School of Medicine, Tehran University of Medical Sciences, Tehran, Iran; 5 Department of Obstetrics and Gynecology, Yas Hospital Complex, Tehran University of Medical Sciences, Tehran, Iran

**Keywords:** Poor ovarian response, Diminished ovarian reserve, Astaxanthin, Oxidative stress, Inflammation, Cell-free DNA, ART outcomes

## Abstract

**Background:**

Poor ovarian response (POR) to controlled ovarian stimulation (COS) remains challenging, especially in advanced-age women with diminished ovarian reserve, resulting in low live birth rates. Many patients prefer to conceive with their eggs, underscoring the need for improved treatments. This study explores astaxanthin potential as a COS adjuvant to improve ovarian response and assisted reproductive technology (ART) outcomes, considering its impact on oxidative stress (OS), inflammation, and apoptosis, which are key factors in POR.

**Methods:**

In this randomized, triple-blind, placebo-controlled trial, 60 infertile POR patients from POSEIDON Group 4 (the poorest prognosis category, age > 35 and poor ovarian reserve (anti-müllerian hormone < 1.2 ng/ml or antral follicle count < 5) undergoing intracytoplasmic sperm injection were enrolled. Patients were assigned to receive either 12 mg/day AST or placebo for eight weeks. All patients underwent a gonadotropin-releasing hormone antagonist regimen for COS. ART outcomes were compared between groups. Blood serum and follicular fluid (FF) were analyzed for OS markers (superoxide dismutase [SOD], total antioxidant capacity [TAC], and malondialdehyde [MDA]), and pro-inflammatory cytokines (interleukin-6 [IL-6], interleukin-8 [IL-8], and vascular endothelial growth factor [VEGF]) via enzyme-linked immunosorbent assay kits, and cell-free DNA [cfDNA] (apoptotic marker) via ALU quantitative polymerase chain reaction.

**Results:**

After the intervention, the AST group exhibited a significant elevation in serum (*P* = 0.013) and TAC (*P* = 0.030), accompanied by a significant reduction in serum MDA (*P* = 0.005). No significant differences between AST and placebo groups were observed in OS markers in FF. AST group showed significant reductions in the serum IL-6 (*P* < 0.001), IL-8 (*P* = 0.001), and VEGF (*P* = 0.002) levels following AST therapy. In the AST group, FF levels of IL-6 (*P* = 0 < 001), IL-8 (*P* = 0.036), VEGF (*P* = 0.006), and cfDNA (*P* < 0.001) were significantly lower than in the placebo group. Between-group comparisons showed significant differences in the alterations of serum SOD (*P* = 0.027), IL-6 (*P* < 0.001), and IL-8 (*P* = 0.035) levels between AST and placebo groups. The AST group showed significant increases in the number of retrieved oocytes (*P* = 0.003), MII oocytes (*P* = 0.004), frozen embryos (*P* = 0.037), and high-quality embryos (*P* = 0.014) compared to the placebo group.

**Conclusion:**

AST shows promise as a COS adjuvant therapy, potentially enhancing some ART outcomes in POR through alleviating OS, inflammation, and apoptosis.

**Trial registration:**

Clinical trial registration number: IRCT20230223057510N1, URL: https://irct.behdasht.gov.ir/trial/68870, registration date: 2023 March 16.

**Graphical Abstract:**

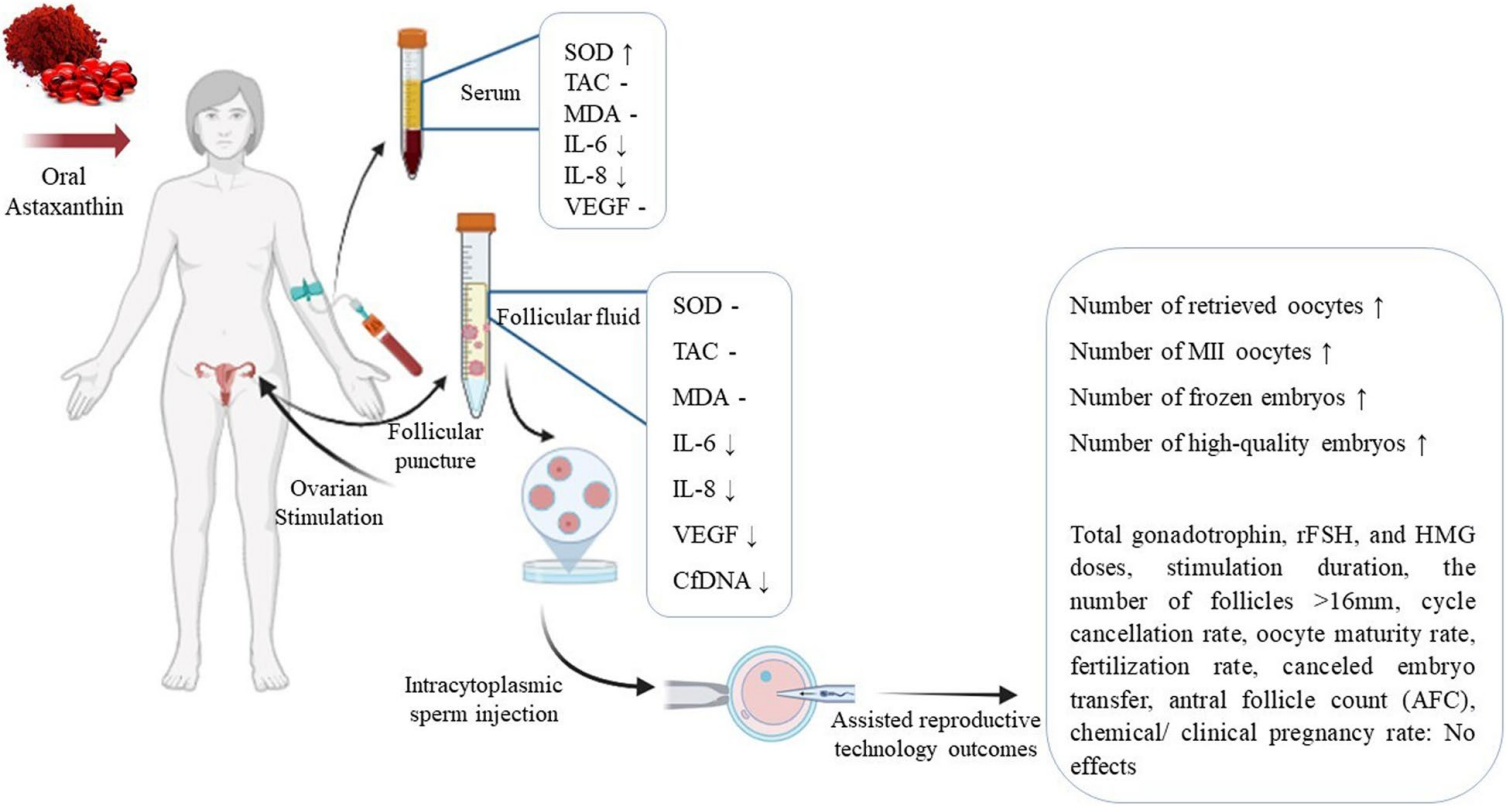

## Introduction

Poor ovarian response (POR) poses a significant dilemma in the treatment of female infertility [1]. Its prevalence, estimated to be between 9% and 24%, is on the rise [2]. The success of assisted reproductive technology (ART) depends on the quantity of retrieved oocytes, and an optimal response involves developing 15 follicles [3, 4]. However, inadequate gonadotropin response during controlled ovarian stimulation (COS) results in poorer ART outcomes compared to normal ovarian responders [5]. According to the POSEIDON stratification, POR is characterized not only by a low number of retrieved oocytes but also by accounting for patient age, ovarian reserve markers (anti-müllerian hormone (AMH) or antral follicle count (AFC)), and previous ovarian response to stimulation. This classification aims to identify patients at risk of poor response better and personalize treatment strategies [6].

Approximately one-third of patients with diminished ovarian reserve (DOR) encounter a poor response to COS [7]. Despite extensive research, POR’s precise pathophysiological mechanisms remain elusive. Studies suggest factors such as oxidative stress (OS), inflammation, and follicular atresia/apoptosis as potential contributors to its pathogenesis [8–11]. To begin with, emerging evidence highlights the role of OS-induced ovarian aging in the development of POR. Notably, POR serves as an early indicator of this aging process. OS can result in damage to vital intracellular macromolecules such as proteins, lipids, and DNA [12]. Additionally, disruption of pro-inflammatory cytokines and growth factors can adversely affect the interaction between FSH (Follicle-stimulating hormone) and its receptor (Follicle-stimulating hormone receptor (FSHR)), resulting in impaired proliferation and differentiation of granulosa cells (GCs) [9], crucial for oocyte development, ovulation, fertilization, and ROS accumulation [13]. Lastly, elevated ROS levels damage cellular components and trigger cell death processes like apoptosis. Proper apoptosis regulation is vital for cellular homeostasis [14]. Ovarian cell apoptosis plays a vital role in the occurrence of extensive follicular atresia or regression, serving as a key mechanism in the process of ovarian aging. Oocyte apoptosis causes germ cell loss, while GCs apoptosis results in nutrient deprivation and metabolic disorders within the ovarian microenvironment [15]. Both contribute to declining ovarian function, with higher GC apoptosis observed in POR patients [16]. Cell-free DNA (cfDNA) refers to fragments of DNA that are derived from cellular apoptosis or necrosis and can be detected in various bodily fluids [17], including follicular fluid (FF) [18]. Increased cfDNA level serve as valuable non-invasive biomarker for early detection and prognosis in cancers and severe diseases [19]. The level of cfDNA in FF serves as an indicator of the proportion of apoptotic and necrotic cells within ovarian follicles [18]. Given that the composition of FF plays a crucial role in oocyte development and the quality of subsequent embryos, heightened levels of cfDNA in FF can have detrimental effects on the development of oocytes and embryos, potentially resulting in unsuccessful ART outcomes [20]. Women with poor ovarian reserve exhibit high concentrations of cfDNA in FF [21], which is attributed to the accelerated apoptosis in the ovary [22]. In light of the discussed content, the quantification of cfDNA in FF presents a non-invasive means to evaluate the quality of the follicular microenvironment.

In spite of various attempted stimulation protocols and treatments for POR, significant improvements in ART outcomes have been elusive [23]. Live birth rates (LBR) for POR patients remain below 10%. While egg donation yields better outcomes, a significant majority of POR patients still desire to conceive using their own eggs. This persistence in seeking conception with their eggs, despite the limited success of certain treatment methods [23, 24] and the complex and time-sensitive nature of their condition [1], highlights the urgent need for a more effective and tailored solution for POR patients.

POR to COS drugs remains a challenge in infertility treatment, especially in advanced age and diminished ovarian reserve women. Recent interest in adjuvant treatment strategies for POR has grown [25–27]. Astaxanthin [3,3′-dihydroxy-β, β′-carotene-4,4′-dione, (AST)], a xanthophyll carotenoid known as the “king of antioxidants,” offers promise due to its multifaceted benefits [28, 29]. It has shown exceptional efficacy, surpassing Coenzyme Q10 (CoQ10), Alpha-Lipoic Acid, and vitamin C [30]. Studies highlight its antioxidant, anti-inflammatory, and anti-apoptotic properties [29]. AST activates the Nrf2/HO-1 pathway, enhancing antioxidant enzymes [31]. Because of its unique structure, ATX quenches singlet oxygen, scavenges free radicals, and inhibits lipid peroxidation, safeguarding membranes [32]. It reduces pro-inflammatory cytokines via NF-κB and MAPK pathways [33]. Recent findings suggest AST’s interplay between Nrf2 and NF-κB pathways, where Nrf2 activation antagonizes NF-κB, potentially leading to anti-inflammatory responses [34]. It also lessens apoptosis through ERK, NF-κB, and PI3K/Akt pathways [35, 36].

This study aims to investigate astaxanthin’s therapeutic potential as a COS adjuvant on ovarian response and assisted reproductive technology (ART) outcomes. Additionally, we will assess astaxanthin’s impact on OS, inflammation, and apoptotic markers.

## Methods

### Trial design

This clinical trial was a prospective, parallel, randomized, triple-blind, and placebo-controlled study. The trial recruited 60 infertile patients with POR undergoing ART at Omid Fertility Clinic in Tehran, Iran, between April and August 2023. Using POSEIDON (Patient-Oriented Strategies Encompassing Individualized Oocyte Number) criteria, the investigation defined POR. The POSEIDON criteria subdivide poor responders into four groups, offering a more detailed and personalized classification of POR by considering age, ovarian reserve markers (Anti-müllerian hormone (AMH) and Antral follicle count (AFC)), and previous response to stimulation. In contrast, the Bologna criteria provide a broader and less specific classification. This approach allows for tailored treatment plans. We included only POSEIDON Group 4 patients in our study because this group represents the poorest prognosis category. By focusing on this subgroup, we aimed to investigate the potential benefits of our intervention in the most challenging cases, where improvement in outcomes would be most clinically significant. The inclusion criteria were: age > 35 years, and poor ovarian reserve (anti-müllerian hormone (AMH) < 1.2 ng/ml, or antral follicle count (AFC) < 5) (indicating low prognosis group 4 following the POSEIDON stratification) [6]. We excluded participants who had ovarian surgery or chemotherapy, endocrine disorders (such as diabetes, thyroid disease, polycystic ovary syndrome (PCOS), hyperprolactinemia), autoimmune disorders (such as the presence of anti-thyroid antibodies), endometriosis, recurrent spontaneous abortion, chromosomal abnormalities, uterine cavity abnormalities, tubal disorders, pelvic inflammatory disease, chronic infectious diseases, cancer, undergone more than three previous ART cycles, received hormone treatment or used intrauterine devices in the past three months, received treatment with dietary supplements and vitamins in the last three months, concurrent severe male factor infertility (notably non-obstructive azoospermia), or spontaneous pregnancy during the intervention. The analysis enrolled POR patients with regular menstrual cycles.

### Ethical approval

This study was performed in accordance with the principles of the Declaration of Helsinki. The trial received approval from the Deputy of the Research and Ethics Committee of TUMS (approval date: 2023 January 01; code: IR.TUMS.MEDICINE.REC.1401.636). Moreover, the protocol was recorded in the Iranian Registry of Clinical Trials (approval date: 2023 March 16; code: IRCT20230223057510N1). Before participating in the study, all participants provided written informed consent.

### Randomization and blinding

**Fig. 1:**
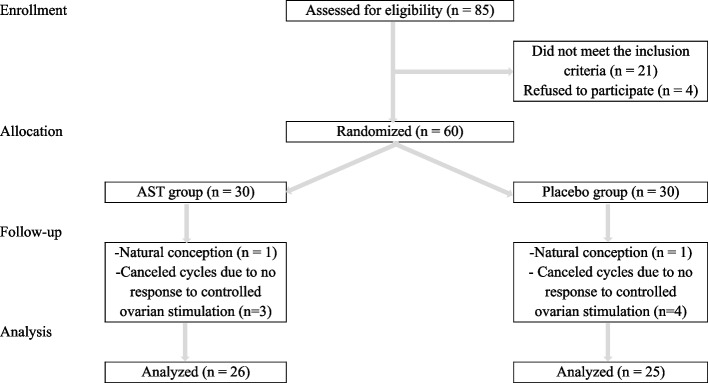
Patient flow across the trial

To ensure unbiased treatment allocation, an independent statistician randomly assigned eligible patients to the AST (*n* = 30) or placebo (*n* = 30) groups in a 1:1 ratio by implementing the balanced block randomization design with a block size of 4. The randomization list was concealed using sequentially numbered, opaque envelopes. This triple-blind trial ensured that patients, researchers, embryologists, laboratory staff, and statisticians were unaware of the individual treatment allocation. It should be emphasized that the AST capsules were indistinguishable from the placebo capsules in size, shape, color, taste, and packaging. In addition, an independent party coded the medicinal content of each bottle with a code unknown to the research team. Also, outcome assessments were conducted by assessors who were completely unaware of participant group assignments. Figure 1 depicts patient flow across the trial in the Consolidated Standards of Reporting Trials (CONSORT) diagram.

### Trial procedures

Infertile patients with POR undergoing intracytoplasmic sperm injection (ICSI) at Omid Fertility Clinic were enrolled if they met the inclusion criteria, which were evaluated by a gynecologist. All patients followed the standard ovarian stimulation with gonadotropin-releasing hormone (GnRH) antagonist. The AST group received 12 mg of oral AST capsules per day (3 × 4 mg capsules; AstaZine^®^, BGG Europe SA., Italy) until the ovum pick-up (OPU) day for eight weeks, while the placebo group received three capsules containing edible paraffin. The intervention duration and dosage were based on an earlier study [37]. Prior studies indicate that consuming 2 to 24 mg of AST daily for at least three weeks offers antioxidant benefits without safety issues or adverse effects [38, 39]. Given the limited number of clinical studies investigating the effects of AST on female infertility and reproductive outcomes [40], our study is innovative, as no clinical trials have specifically explored AST’s effects on POR patients. Additionally, previous research has demonstrated that AST administration improves ART outcomes by increasing the number of mature (MII) oocytes retrieved [41–43]. The dosage and duration of the intervention were based on a previous randomized clinical trial, in which 12 mg/day of AST was administered for 60 days to women with PCOS. That study showed that AST enhances ART outcomes by elevating TAC levels in FF and modulating endoplasmic reticulum (ER) stress in GCs, without negatively affecting MDA or SOD levels, or compromising follicular health [37]. Furthermore, AST intake at this dosage improved serum and FF apoptotic factor levels while modulating the expression of genes and proteins involved in the apoptosis pathway in GCs [44]. These studies also reported no adverse effects on follicular health, confirming that this dosage is safe and appropriate for our investigation. The absence of adverse effects on follicular health further underscores the suitability of this dosage, which we selected based on these promising findings. To monitor medication adherence and potential side effects, patients received weekly calls and monthly visits. Patients were advised to continue with their usual daily routines and to abstain from consuming any dietary supplements. The adherence rate was assessed via returned capsules on OPU day [45].

### Blood and FF collection

Following a prior study [41], 10 ml of venous blood was collected pre- and post-intervention (day of OPU) to assess OS markers and pro-inflammatory cytokines. Serum obtained by centrifugation (1500 g, 10 min) was stored at -80 °C for analysis. To reduce the risk of blood contamination, FF was only collected from the first follicle during the OPU. After centrifugation (3000 g for 15 min), the FF supernatants were filtered using 0.45 μm filters to remove cell debris and stored at -80 °C for later analysis [46]. Pre-intervention serum samples were collected on days 2 or 3 of the follicular phase. Post-intervention serum samples and FF were obtained on the day of OPU, with all samples collected under fasting conditions.

### OS markers and pro-inflammatory cytokines

Serum (pre- and post-intervention) and FF samples were analyzed for OS markers (superoxide dismutase [SOD], total antioxidant capacity [TAC], and malondialdehyde [MDA]) using human enzyme-linked immunosorbent assay (ELISA) kits (Zellbio, GmbH, Germany), and for pro-inflammatory cytokines (interleukin-6 [IL-6], interleukin-8 [IL-8], and vascular endothelial growth factor [VEGF]) using human ELISA kits (Karmania Pars Gene Co.; KPG, Iran). All parameters were blindly measured twice.

### CfDNA

Interestingly, measuring cfDNA levels—derived from apoptosis or necrosis—in FF has become a valuable diagnostic tool for assessing ovarian function [18, 19]. In poor ovarian reserve, higher cfDNA in FF is tied to accelerated ovarian apoptosis [47], adversely affecting ART outcomes [46]. CfDNA was extracted from FF using a previously described method [18, 48]. Quantification of total cfDNA utilized qPCR with ALU 115 primers. Each ALU-qPCR reaction included 1µL of FF, 0.25 µM forward and reverse ALU 115 primers, and 5 µL of 2X SYBR Green I master mix (Amplicon, Denmark). CfDNA levels in FF were measured via a standard curve derived from successive genomic DNA dilutions [48]. Negative controls (no template) were included, and each FF was tested in quadruplicate. To assess the origin of cfDNA, qPCR with ALU 247 primers was employed to quantify necrosis-related fragments. The Q247/Q115 ratio, indicating the proportion of cfDNA generated from necrosis over total cfDNA, was used to calculate cfDNA integrity [46]. If the cfDNA integrity falls below 0.5, it is primarily associated with apoptotic events; otherwise, it is predominantly linked to necrotic events. This study employed the following primers: ALU115 forward (5’-CCTGAGGTCAGGAGTTCGAG-3’), ALU115 reverse (5’-CCCGAGTAGCTGGGATTACA-3’), ALU247 forward (5’-GTGGCTCACGCCTGTAATC-3’), and ALU247 reverse (5’-CAGGCTGGAGTGCAGTGG-3’).

### COS protocol

At Omid Fertility Clinic in Tehran, Iran, the combination of a GnRH antagonist protocol and whole embryo freezing has proven to be the most effective method for inducing ovulation in POR patients. On day 2 of the menstrual cycle, a combination of recombinant FSH (rFSH) (225 IU/day, Gonal-F^®^, Merck Serono SA, Switzerland) and human menopausal gonadotrophin (HMG) (FSH 75 IU: LH 75 IU, 300 IU/day, HUMEGNAN^®^, Darou Pakhsh Pharmaceutical Mfg. Co., Iran) was initiated. This was continued up to the human chorionic gonadotropin (hCG) trigger. Repeated transvaginal ultrasound was used to monitor ovarian response and guide dose adjustments. The GnRH antagonist, Cetrorelix acetate (250 µg/day, Cetrotide, Merck Serono SA, Switzerland), was given once 2 or more follicles reached ≥ 14 mm in diameter. Cetrotide was ceased upon the attainment of a diameter of ≥ 18 mm in at least 2 follicles, and the final oocyte maturation was triggered using 10,000 IU hCG (Ovitrelle, Merck Serono SA, Switzerland). If no ≥ 14 mm follicles were observed after 8–9 days, the cycle was canceled. Ultrasound-guided OPU was done 36 h after the trigger. The standard ICSI protocol was employed for all participants. Embryos were cryopreserved on day 3 to enhance clinical outcomes, and 2 or 3 cleavage or blastocyst stage embryos were transferred two cycles later, as per established local clinical practice. When only one embryo was available, a single embryo transfer (SET) was executed.

### ART outcomes

In this study, the cumulus-oocyte complexes were stripped of cumulus cells using hyaluronidase enzyme (Sigma^®^, USA) two hours post the OPU procedure. The oocytes were then evaluated for maturity using a stereo microscope (Olympus SZX7, Tokyo, Japan) and categorized as germinal vesicle (GV), metaphase I (MI), and metaphase II (MII). Suitable MII oocytes were used for the ICSI procedure, and 16–18 h later, fertilization was evaluated by checking for 2 pronuclei (2 PN) and 2 polar bodies. The study collected data from reproductive outcomes, including parameters related to ovarian response (such as total gonadotrophin, rFSH, and HMG doses, stimulation duration, the number of follicles > 16 mm on the day of triggering, and cycle cancellation rate (Percentage of cycles canceled before OPU per the number of started cycles [49]), the number of retrieved oocytes, the number of MII oocytes, oocyte maturity rate (Percentage of normal MII oocytes per total normal retrieved oocytes [49]), fertilization rate (Percentage of oocytes with 2PN/2 PB, 16–18 h post-insemination per injected MII oocytes [50]), the number of frozen embryos, high-quality embryos (number of grade A and B cleavage embryos according to the ASEBIR (Association for the Study of Reproductive Biology) criteria [51]), canceled embryo transfers (ET) due to the absence of usable embryos, chemical pregnancy rate (percentage of the pregnancies with a positive serum b-hCG test 14 days after ET per the number of ET cycles [52]), and clinical pregnancy rate (percentage of pregnancies showing confirmed clinical markers on ultrasound (like gestational sac and heart rate) per the number of ET cycles [49]). Furthermore, the AFC assessment was conducted pre- and post-intervention.

### Sample size and statistical analysis

The sample size calculation was based on the mean number of MII oocytes. According to similar clinical trials [25, 26, 53, 54], the mean number of MII oocytes in POR patients was considered to be about 3.2 ± 2 in the control group. We anticipated a 50% increase in the mean number of MII oocytes in the intervention group. We determined that a sample size of 50 participants (25 in each arm) was required to detect this difference. To account for a 20% dropout rate, the total sample size was adjusted to 60 participants (30 in each group). This sample size ensures an 80% power to detect a significant difference at the 0.05 significance level. The following formula was applied to calculate the sample size:

$$\begin{aligned} k &= \frac{n_{2}}{n_{1}} = 1 \\ n_{1} & = \frac{\left( \sigma^{2}_{1} + \sigma^{2}_{2} / K \right) \left( z_{1-\alpha/2} + z_{1-\beta} \right)^{2}}{\Delta^{2}} \\ n_{1} & = \frac{\left( 2^{2} + 2^{2} / 1 \right) (1.96 + 0.84)^{2}}{1.6^{2}} \\ n_{1} &= 25 \\ n_{2} & = K \ast n_{1} =25 \end{aligned}$$

Δ = |µ_2_-µ_1_| = absolute difference between two means.

σ_1_, σ_2_ = variance of mean #1 and #2

n_1_ = sample size for group #1

n_2_ = sample size for group #2

α = probability of type I error (usually 0.05)

β = probability of type II error (usually 0.2)

z = critical Z value for a given α or β

k = ratio of sample size for group #2 to group #1

Quantitative variables were presented as mean ± standard deviation (SD), and qualitative variables were presented as numbers and percentages. Qualitative variables were compared between the AST and placebo groups using Fisher’s exact and Pearson’s chi-squared tests. The distribution of data was assessed using the Shapiro-Wilk test. To compare continuous variables with a normal distribution between the AST and placebo groups, an independent sample t-test was applied. Non-normally distributed data were analyzed using the Mann-Whitney U test. Pre- and post-intervention markers within each group were compared using Student’s paired t-test. Analysis of medication effectiveness between two groups was performed using a repeated-measures analysis of variance (ANOVA) model. Treatment and time effects and also the interaction between time and treatment (time*group) were included in this model. Logistic regression was employed to assess ART outcomes, with age, AMH, and AFC included as confounding variables, as they are key markers of ovarian response to COS. Statistical significance is denoted by a *P*-value of less than 0.05.

## Results

### Baseline characteristics

Presented in the CONSORT flowchart, the study encompassed the randomization and allocation of 60 patients. Ultimately, 51 participants from this cohort were included in the final analysis (AST group: *n* = 26, placebo group: *n* = 25) (Fig. 1). Importantly, no adverse effects or instances of toxicity were reported by the patients throughout the intervention. At the study’s inception, no significant differences emerged in age, BMI, infertility duration, menstrual cycle duration, hormonal profile, AFC, and adherence rates between the two groups (Table 1). Primary infertility was diagnosed in the majority of patients in both groups. Additionally, the predominant stage of embryos transferred in both the AST and placebo groups was the blastocyst stage.

**Table 1:** Baseline characteristics of the participants

**Variables**	AST (*n* = 26)	Placebo (*n* = 25)	*P*-value
**Age (years)**	38.42 ± 1.79	38.32 ± 1.67	0.811
**BMI (kg/m2)**	22.59 ± 1.25	22.46 ± 1.53	0.729
**Infertility duration (years)**	3.31 ± 1.25	2.88 ± 1.42	0.223
**Primary infertility, n (%)**	20/26 (76.9%)	17/25 (68.0%)	0.475
**Menstrual cycle duration**	26.96 ± 3.38	27.44 ± 3.64	0.629
**AMH (µg/mL)**	0.75 ± 0.16	0.71 ± 0.21	0.368
**FSH (mIU/mL)**	9.87 ± 1.76	9.95 ± 2.11	0.880
**LH (mIU/mL)**	4.76 ± 1.17	4.56 ± 1.09	0.523
**E2 (pg/mL)**	71.63 ± 26.14	68.27 ± 21.89	0.678
**P4 (ng/mL)**	0.59 ± 0.37	0.53 ± 0.39	0.421
**PRL (mIU/L)**	200.15 ± 34.50	186.56 ± 28.37	0.132
**AFC (n)**	4.38 ± 1.41	4.12 ± 1.166	0.365
**Adherence rate (%)**	98.80 ± 2.01	98.73 ± 2.20	0.945
**Blastocyst stage embryo transfer (%)**	18/24 (75%)	15/23 (65.2%)	0.464

*AST* astaxanthin group, *BMI* body mass index [weight (kg)/height (m2)], *AMH* anti-müllerian hormone, *FSH* follicle-stimulating hormone, *LH* luteinizing hormone, *E2* estradiol, *P4* progesterone, *PRL* prolactin, *AFC* antral follicle count, Adherence rate (Number of dosage units dispensed − number of dosage units remained)/ (prescribed number of dosage unit per day × number of days between 2 visits), *n* number. Table values represent either mean ± *SD* or number (percentages). Statistical significance is denoted by a *P*-value of less than 0.05

### Serum and FF OS markers and pro-inflammatory cytokines

At baseline, the serum levels of markers displayed no significant differences between the AST and placebo groups. After the intervention, the AST group exhibited a significant increase in serum SOD (*P* = 0.013) and TAC (*P* = 0.030), along with a significant reduction in serum MDA (*P* = 0.005). However, the placebo group showed no significant changes in marker levels after the intervention. Between-group comparisons showed a statistically significant difference in the alterations of serum SOD levels between the AST and placebo groups (*P* = 0.027). No significant differences were observed in alterations of serum levels of TAC (*P* = 0.246) and MDA (*P* = 0.261) between the AST and placebo groups (Table 2). The FF levels of SOD (*P* = 0.607), TAC (*P* = 0.792), and MDA (*P* = 0.887) exhibited no significant differences when comparing the AST group to the placebo group (Table 3).

**Table 2:** Comparison of pre- and post-intervention serum levels of OS markers and inflammatory cytokines between AST and placebo groups

	AST (*n* = 26)			Placebo (*n* = 25)			
**Variables**	Pre-intervention serum levels	Post-intervention serum levels	Paired *P*-value	Pre-intervention serum levels	Post-intervention serum levels	Paired *P*-value	*P*-value between groups
**SOD (U/ml)**	14.97 ± 2.26	16.26 ± 2.22	0.013^*^	15.28 ± 2.01	14.59 ± 3.59	0.351	0.027^*^
**TAC (µmol/L)**	892.50 ± 81.77	910.05 ± 92.56	0.030^*^	918.73 ± 127.35	889.06 ± 203.20	0.468	0.246
**MDA (µmol/L)**	16.82 ± 1.22	16.19 ± 1.27	0.005^*^	16.74 ± 1.44	16.43 ± 1.64	0.135	0.261
**Il-6 (pg/ml)**	1.74 ± 0.33	1.25 ± 0.32	0.000^*^	1.61 ± 0.33	1.67 ± 0.30	0.098	0.000^*^
**Il-8 (pg/ml)**	14.62 ± 1.87	13.38 ± 1.65	0.001^*^	14.58 ± 2.22	14.28 ± 2.55	0.323	0.035^*^
**VEGF (pg/ml)**	13.20 ± 2.96	12.37 ± 2.87	0.002^*^	12.71 ± 3.19	12.43 ± 3.20	0.123	0.071

*AST* Astaxanthin group, *SOD* superoxide dismutase, *TAC* total antioxidant capacity, *MDA* malondialdehyde, *IL-6* interleukin 6, *IL-8* interleukin 8, *VEGF* vascular endothelial growth factor. Table values represent mean ± *SD*. Statistical significance is denoted by a *P*-value of less than 0.05 (*P < 0.05)

**Table 3:** Comparison of FF levels of OS markers, inflammatory cytokines, and cfDNA between AST and placebo groups

**Variables**	AST (*n* = 26), FF levels	Placebo (*n* = 25), FF levels	*P*-value
**SOD (U/ml)**	14.49 ± 2.12	14.83 ± 2.60	0.607
**TAC (µmol/L)**	816.18± 126.06	835.71 ± 149.95	0.792
**MDA (µmol/L)**	12.94 ± 1.16	13.32 ± 2.05	0.887
**Il-6 (pg/ml)**	2.05± 0.60	2.98 ± 1.02	0.000^*^
**Il-8 (pg/ml)**	24.52 ± 3.68	28.66 ± 8.74	0.036^*^
**VEGF (pg/ml)**	11.04 ± 2.06	13.16 ± 3.02	0.006^*^
**CfDNA level (ALU115) (ng/µl)**	0.39± 0.12	0.60± 0.17	0.000^*^
**ALU247 (ng/µl)**	0.18± 0.07	0.21± 0.07	0.133
**CfDNA integrity (ALU 247/ ALU 115)**	0.48±0.14	0.38±0.13	0.014^*^

*AST* astaxanthin group, *SOD* superoxide dismutase, *TAC* total antioxidant capacity, *MDA* malondialdehyde, *IL-6* interleukin 6, *IL-8* interleukin 8, *VEGF* vascular endothelial growth factor, *cfDNA* Cell-free DNA, *FF* follicular fluid. Table values represent mean ± *SD*. Statistical significance is denoted by a *P*-value of less than 0.05 (*P < 0.05)

The results of pro-inflammatory cytokines indicated significant reductions in serum IL-6 (*P* < 0.001), IL-8 (*P* = 0.001), and VEGF (*P* = 0.002) following AST therapy in the AST group. However, no significant changes in cytokines levels were observed in the placebo group. Between-group comparisons showed statistically significant differences in the alterations of serum IL-6 and IL-8 levels between the groups (*P* < 0.001 and *P* = 0.035, respectively). No significant difference was observed in the alteration of VEGF (*P* = 0.071) between the AST and placebo groups (Table 2). Additionally, in the AST group, FF levels of IL-6 (*P* = 0 < 001), IL-8 (*P* = 0.036), and VEGF (*P* = 0.006) were significantly lower than in the placebo group (Table 3).

### FF cfDNA

AST supplementation significantly reduced cfDNA levels, as measured by ALU115 qPCR (*P* < 0.001), without affecting ALU247 levels (*P* = 0.133) compared to the placebo group. Furthermore, cfDNA integrity significantly increased in the AST group compared to the placebo group (*P* = 0.014). The mean Q247/Q115 ratio in FF samples was 0.48 ± 0.14 in the AST group and 0.38 ± 0.13 in the placebo group, suggesting that the analyzed cfDNA predominantly originates from cellular apoptosis.

### Ovarian stimulation parameters and ART outcomes

The AST group showed a significant increase in the number of retrieved oocytes (*P* = 0.003), MII oocytes (*P* = 0.004), frozen embryos (*P* = 0.037), and high-quality embryos (*P* = 0.014) compared to the placebo group. Notably, other factors such as total gonadotrophin (*P* = 0.207), rFSH (*P* = 0.149), and HMG doses (*P* = 0.299), stimulation duration (*P* = 0.149), the number of follicles > 16 mm (*P* = 0.186), oocyte maturity rate (*P* = 0.089), fertilization rate (*P* = 0.973), canceled ET (*P* = 0.682), chemical pregnancy rate (*P* = 0.995), and clinical pregnancy rate (*P* = 0.695), showed no significant changes between the AST and placebo groups (Table 4). Also, The cycle cancellation rate showed no significant difference between the AST group (3/29, 10.3%) and the placebo group (4/29, 13.8%) (*P* = 1.000). Furthermore, the pre- and post-intervention changes in AFC did not significantly differ between the AST (4.38 ± 1.41 vs. 4.42 ± 1.33; *P* = 0.788) and placebo groups (4.12 ± 1.16 vs. 4.00 ± 1.15; *P* = 0.083) (*P*-value between groups = 0.321).

**Table 4:** Comparison of ART cycle stimulation parameters, embryology, and clinical reproductive outcomes between AST and placebo groups

**Variables**	AST (*n* = 26)	Placebo (*n* = 25)	*P*-value^#^
**Total gonadotrophin doses (IU)**	5648.08 ± 623.816	5862.00 ± 653.00	0.207
**rFSH doses (IU)**	2423.08 ± 232.155	2520.00 ± 243.02	0.149
**HMG dose (IU)**	3225.00 ± 453.486	3342.00 ± 427.61	0.299
**Stimulation duration (days)**	10.77 ± 1.03	11.20 ± 1.08	0.149
**Number of follicles >16mm**	4.42 ± 1.33	3.96 ± 1.13	0.186
**Retrieved oocytes (n)**	4.38 ± 1.35	3.36 ± 1.03	0.003^*^
**GV (n)**	0.73 ± 0.60	0.88 ± 0.60	0.188
**MI (n)**	0.27 ± 0.45	0.32 ± 0.47	0.723
**MII (n)**	3.38 ± 1.44	2.16 ± 0.89	0.004^*^
**Oocyte maturity rate (MII %)**	75.64 ± 17.03	66.00 ± 22.02	0.089
**Fertilization rate (%)**	89.74 ± 15.33	90.33 ± 16.25	0.973
**Frozen embryos (n)**	2.73 ± 1.61	1.80 ± 0.86	0.037^*^
**High-quality embryos (n)**	2.62 ± 1.41	1.64 ± 0.75	0.014^*^
**Transferred embryos (n)**	1.73 ± 0.66	1.68 ± 0.74	0.776
**Canceled ET, n (%)**	2/26 (7.7%)	2/25 (8.0%)	0.682
**Chemical pregnancy rate, n (%)**	9/24 (37.5%)	8/23 (34.8%)	0.995
**Clinical pregnancy rate, n (%)**	5/24 (20.8%)	4/23 (17.4%)	0.695

*AST *Astaxanthin group, *rFSH *recombinant follicle-stimulating hormone, *HMG *human menopausal gonadotropin, *GV* germinal vesicle, *MI* metaphase I, *MII *metaphase II, *ET* embryo transfer, *n* Number. Table values represent either mean ±*SD* or number (percentages)^. #^Logistic regression adjusted for age, *AMH*, and *AFC *as confounding variables. Statistical significance is denoted by a*P*-value of less than 0.05 (*P < 0.05)

## Discussion

The pathophysiology of POR primarily stems from the limited number of follicles that respond to COS [55]. This leads to fewer retrieved oocytes, lower chances of conception, and a higher risk of cycle cancellation [56]. OS, inflammation, and apoptosis are key contributors to this condition [8–11]. OS, in particular, accelerates ovarian aging and damages critical macromolecules, impairing oocyte quality, embryo development, and implantation [57]. Pro-inflammatory cytokines disrupt the function of GCs, which are crucial for oocyte maturation, further exacerbating POR through impaired FSH signaling [9]. Elevated levels of ROS also promote apoptosis in oocytes and GCs, worsening ovarian function [14]. While various stimulation protocols and adjuvant treatments have been explored, significant improvements in ART outcomes for POR patients remain limited [56]. Astaxanthin, with its potent antioxidant, anti-inflammatory, and anti-apoptotic properties, may help improve POR [29]. To explore this, we conducted a randomized, triple-blind, placebo-controlled trial with POR patients, assessing astaxanthin’s role as a COS adjuvant on ovarian response, ART outcomes, and markers of OS, inflammation, and apoptosis.

Antioxidant supplementation holds considerable promise in mitigating the detrimental effects of OS on oocyte and embryo quality in patients with POR [8, 58–60]. In this trial, AST therapy demonstrated remarkable advancements in oocyte quantity and maturity, the number of frozen embryos, and high-quality embryos. Our study results suggest that AST has the potential to enhance the FF quality, a critical factor in oocyte development. We hypothesized that mitigating OS, reducing inflammation, and inhibiting apoptosis, all linked to POR, could achieve this improvement. Consequently, these effects likely result in the production of higher-quality oocytes, subsequently leading to the development of high-quality embryos and an overall improvement in other ART outcomes. On the other hand, the fertilization rate between the two groups was similar. It’s important to note that even when excluding male factor patients, sperm quality can still influence the fertilization process. No significant differences were observed in ovarian response parameters, oocyte maturity rate, canceled ET, pregnancy outcomes, and AFC. Further research with a larger sample size is warranted to explore the impact of varying AST dosages and treatment durations, which could potentially yield different outcomes regarding astaxanthin’s effectiveness. The findings from this study are consistent with our prior clinical trials involving PCOS and endometriosis patients [37, 41]. Similar results were seen with CoQ10 therapy in a trial for POR patients [26].

In our trial, serum levels of all OS markers were improved in the AST group. There was a statistically significant difference in the alterations of serum SOD levels between the groups, but not in TAC and MDA levels. However, FF levels of OS markers did not change between the two groups. Results align with earlier RCTs, showing AST supplementation’s positive impact on OS in overweight/obese individuals [61, 62]. Our previous studies demonstrated increased TAC levels in PCOS patients’ FF, while SOD and MDA showed no significant changes following AST supplementation [37]. Likewise, in endometriosis patients, AST elevated serum SOD and TAC levels and reduced MDA, but no significant changes in OS markers were observed in the FF [41].

Notably, AST was found to significantly decrease IL-6, IL-8, and VEGF levels both in the serum and FF of the AST group. There were statistically significant differences in the alterations of serum IL-6 and IL-8 levels between the groups, but not in VEGF levels. Reports indicate that IL-6 reduces follicular aromatase activity, leading to lower E2 levels and negatively affecting fertility and fertilizing capacity [8]. Additionally, IL-8 attracts and activates leukocytes and macrophages, intensifying OS by promoting ROS production [63]. Schafer et al.’s findings support a positive association between OS and VEGF gene expression [64]. Our study concurs with earlier research, highlighting AST’s ability to reduce pro-inflammatory cytokine levels in the serum and FF of endometriosis patients [41]. Meanwhile, AST has demonstrated the ability to regulate essential pro-inflammatory factors, including IL-1β, IL-6, IL-8, VEGF, and TNF-α [65, 66]. In 2016, Nuñez-Calonge et al. reported increased OS markers and pro-inflammatory cytokines (IL-6, IL-8, VEGF) in FF of low ovarian response patients, along with reduced antioxidant enzyme activity [8]. Taghavi et al. also observed elevated IL-6 and IL-8 levels in POR women compared to normal responders [9]. However, our findings highlight the potential of AST in promoting antioxidant balance and combating OS and inflammation associated with POR

In the current study, the observed significant reduction in cfDNA levels (ALU115) and the significant increase in cfDNA integrity, accompanied by an unchanged ALU247 level (a marker of necrosis) following AST therapy, suggest that AST’s anti-apoptotic effects may contribute to these changes. OS-induced apoptosis in follicles and GCs can lead to the release of cfDNA into the FF [46, 67]. Also, apoptosis-induced cell debris continues to affect the ovarian microenvironment by increasing cfDNA levels, which stimulates intracellular ROS production and intensifies apoptosis [68]. The association between cfDNA levels in FF and ovarian reserve is of notable significance. Moreover, increased cfDNA in FF is linked to POR, and reduced oocyte and embryo quantity/quality, leading to poor ART outcomes [46, 68]. In a 2019 study by Nagireddy et al., serum cfDNA levels in low ovarian responders correlated positively with FSH levels and negatively with AFC and AMH levels [69]. In our 2021 experiment, AST reduced OS and showed a modest decrease in the rate of apoptosis in the GCs of a PCOS mouse model, and activated the PI3K/AKT pathway [70]. Besides, in a 2023 clinical trial, AST suppressed GCs apoptosis triggered by endoplasmic reticulum stress in PCOS patients [37]. Indeed, the evidence consistently confirms AST’s capacity to inhibit apoptosis induced by OS [71]. This aligns with the recent trial in POR patients, where AST demonstrated the ability to decrease cfDNA levels in FF, possibly by countering OS-induced apoptosis through its antioxidant and anti-apoptotic properties. However, additional research is needed to assess cfDNA levels in serum following AST therapy.

Given the limited number of clinical studies investigating the effects of AST on female infertility and reproductive outcomes [40], our study is innovative, as no clinical trials have specifically explored AST’s effects on POR patients. To the best of our knowledge, this marks the first randomized, triple-blind, placebo-controlled clinical trial investigating the impact of AST supplementation on OS markers, pro-inflammatory cytokines, cfDNA, and ART outcomes in POR patients. Our research’s strength lies in assessing a homogeneous (Group 4, according to the POSEIDON stratification, representing the poorest prognosis category), nationwide group of POR patients who underwent consistent treatment procedures. In addition, we chose cfDNA as an apoptosis biomarker in FF due to its strong link to cellular death. Moreover, cfDNA offers several advantages over other apoptotic markers, including its stability, ease of quantification, and potential as a non-invasive biomarker. Furthermore, FF cfDNA may serve as a novel indicator of follicular microenvironment quality [46]. It is pertinent to highlight that our study had some limitations. To achieve a more robust analysis of pregnancy outcomes, a larger sample size is imperative. Unfortunately, we were unable to evaluate the LBR, a crucial ART success indicator, due to time constraints. Additionally, the study did not assess serum cfDNA levels and focused on frozen ICSI cycles.

## Conclusion

In conclusion, AST demonstrates promise as an adjuvant therapy in COS for patients with POR. Our findings suggest that AST supplementation may improve ART outcomes, including an increased number of retrieved oocytes, MII oocytes, frozen embryos, and high-quality embryos. These improvements are likely mediated through the mitigation of OS (evidenced by elevated SOD and TAC levels and reduced MDA), the reduction of inflammatory markers (IL-6, IL-8, VEGF), and the suppression of apoptotic activity (as indicated by decreased cfDNA levels).

## Data Availability

No datasets were generated or analysed during the current study.

## Abbreviations

POR: Poor ovarian response
ART: Assisted reproductive technology
COS: Controlled ovarian stimulation
OS: Oxidative stress
FSH: Follicle-stimulating hormone
FSHR: Follicle-stimulating hormone receptor
GCs: Granulosa cells
ROS: Reactive oxygen species
cfDNA: Cell-free DNA
FF: Follicular fluid
LBR: Live birth rates
AST: Astaxanthin
CoQ10: Coenzyme Q10
Nrf2/HO-1: Nuclear factor erythroid 2–related factor 2/ heme oxygenase-1
NF-κB: Nuclear factor kappa B
MAPK: Mitogen-activated protein kinase family
ERK: Extracellular signal-regulated kinase
PI3K/Akt: Phosphoinositide 3-kinase/protein kinase B
AMH: Anti-müllerian hormone
AFC: Antral follicle count
POSEIDON: Patient-Oriented Strategies Encompassing Individualize Oocyte Number) stratification
PCOS: Polycystic ovary syndrome
CONSORT: Consolidated Standards of Reporting Trials
ICSI: Intracytoplasmic sperm injection
GnRH: Gonadotropin-releasing hormone
OPU: Ovum pick-up
SOD: Superoxide dismutase
TAC: Total antioxidant capacity
MDA: Malondialdehyde
IL-6: Interleukin-6
IL-8: Interleukin-8
VEGF: Vascular endothelial growth factor
ELISA: Enzyme-linked immunosorbent assay
rFSH: Recombinant FSH
HMG: Human menopausal gonadotrophin
hCG: Human chorionic gonadotropin
SET: Single embryo transfer
GV: Germinal vesicle
MI: Metaphase I
MII: Metaphase II
2 PN: 2 pronuclei
ASEBIR: Association for the Study of Reproductive Biology
ET: Embryo transfers
SD: Standard deviation
ANOVA: Analysis of variance
